# A novel lncRNA‐miRNA‐mRNA competing endogenous RNA regulatory network in lung adenocarcinoma and kidney renal papillary cell carcinoma

**DOI:** 10.1111/1759-7714.14129

**Published:** 2021-08-28

**Authors:** Qiwei Zhou, Diangeng Li, Hongying Zheng, Zheng He, Feng Qian, Xiaotian Wu, Zhiwei Yin, Peng Tao Bao, Meiling Jin

**Affiliations:** ^1^ Department of Urology Chinese People's Liberation Army General Hospital/PLA Medical School Beijing China; ^2^ Department of Urology Chinese People's Liberation Army No.92493 Hospital Huludao China; ^3^ Department of Scientific Research Beijing‐Chaoyang Hospital Beijing China; ^4^ Harbin Medical University Ha'erbin China; ^5^ Department of Laboratory Chinese People's Liberation Army General Hospital Beijing China; ^6^ Department of Emergency Medicine Chinese People's Liberation Army No. 92493 Hospital Huludao China; ^7^ College of Integration Science, Yanbian University Yanbian China; ^8^ Department of Nephrology Chinese People's Liberation Army General Hospital, Chinese People's Liberation Army Institute of Nephrology, State Key Laboratory of Kidney Diseases (2011DAV00088), National Clinical Research Center for Kidney Diseases Beijing China; ^9^ Department of Respiratory Medicine The Eighth Medical Center of Chinese People's Liberation Army General Hospital Beijing China; ^10^ Department of Nephrology Beijing‐Chaoyang Hospital Beijing China

**Keywords:** competing endogenous RNAs, GPRIN1, Kidney renal papillary cell carcinoma, Lung adenocarcinoma, miR‐140‐3p

## Abstract

**Background:**

GPRIN1 may be a novel tumor regulator, but its role and mechanism in tumors are still unclear.

**Methods:**

First, a pan‐cancer correlation analysis was conducted on the expression and prognosis of GPRIN1 based on the data downloaded from The Cancer Genome Atlas (TCGA) database. Second, the Starbase database was used to predict the upstream miRNAs and lncRNAs of GPRIN1, and the expression analysis, survival analysis, and correlation analysis were performed to screen the microRNA (miRNAs)/long non‐coding RNAs (lncRNAs) that had a correlation with kidney renal papillary cell carcinoma (KIRP) or lung adenocarcinoma (LUAD). Third, the CIBERSORT algorithm was employed to calculate the proportion of various types of immune cells, and then the R packages were used for evaluating the relation between GPRIN1 expression and tumor immune cell infiltration as well as between GPRIN1 and the immune cell biomarker. Finally, the correlation analysis was made on GPRIN1 and immune checkpoints (CD274, CTLA4, and PDCD1).

**Results:**

The pan‐cancer analysis suggested that GPRIN1 was up‐expressed in KIRP and LUAD, and it correlated with poor prognosis. LINC00894/MMP25‐AS1/SNHG1/LINC02298/MIR193BHG‐miR‐140‐3p was likely to be the most promising upstream regulation pathway of GPRIN1. Upexpression of LINC00894/MMP25‐AS1/SNHG1/LINC02298/MIR193BHG and downexpression of miR‐140‐3p were found relevant with poor outcomes of KIRP and LUAD. GPRIN1 expression was significantly correlated with tumor immune cell infiltration, immune cell biomarkers, and immune checkpoints.

**Conclusions:**

The competitive endogenous (ceRNA) of miR‐140‐3p‐GPRIN1 axis and its upstream lncRNAs are closely related to KIRP and LUAD, and might affect the prognosis and therapeutic effect of KIRP and LUAD.

## Graphical abstract

GPRIN1 is an important prognostic marker for KIRP and LUAD. ncRNA (LINC00894/MMP25‐AS1/SNHG1/LINC02298/MIR193BHG)‐mediated high expression of GPRIN1 correlates with poor prognosis and tumor immune infiltration of KIPR and LUAD.

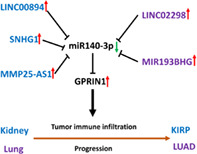

## INTRODUCTION

The G protein regulated inducer of neurite outgrowth 1 (GPRIN1) is a protein coding gene. Diseases associated with GPRIN1 include cerebral creatine deficiency syndrome 2 and cerebral creatine deficiency syndrome, for which Methyl‐CpG Binding Protein 2 (MECP2) and associated Rett syndrome are relatable pathways. Current studies of GPRIN1 are limited and insufficient. The Cancer Genome Atlas (TCGA) project has generated genomic, epigenomic, transcriptomic, and proteomic data for over 20 different cancer types [14–21]. These data sets provide broad insight into the underlying genetic aberrations existing across multiple cancer types. In addition, TCGA has clinical data describing specific metrics such as histopathology and clinical stage, among others. Overall, TCGA data has the potential for determining the clinical significance of critical genetic aberrations.^1^ Pan‐cancer analysis of 33 tumors performed in this study based on the data from TCGA found that GPRIN1 was significantly overexpressed in a variety of tumors together with its correlation with prognosis. Thus, it has been speculated that GPRIN1 might be closely related to tumorgenesis.

In this study, expression analysis and survival analysis of GPRIN1 in various types of tumors were first performed. Then the upstream microRNAs (miRNAs) and long non‐coding RNAs (lncRNAs) of GPRIN1 were predicted to establish the competing endogenous RNAs (ceRNA) action network of lncRNA‐miRNA‐GPRIN1. Finally, assessment was made on the relationship between GPRIN1 and immune cell infiltration, immune cell markers, and immune checkpoints, respectively. GPRIN1 proved to associate with poor prognosis and tumor immune invasion of kidney renal papillary cell carcinoma (KIRP) or lung adenocarcinoma (LUAD).

## METHODS

### Data collection, preprocessing, and analysis

The RNA sequencing transcriptome data and clinical data of 33 cancer types were downloaded from TCGA database (https://portal.gdc.cancer.gov/). Perl script was used to organize the data. The Wilcoxon signed‐rank test was utilized to identify differentially expressed genes by the limma package of R on account of the cutoff values: |Log2FC| > 1 and FDR < 0.05. The survival package of R was applied in identifying the prognostic gene. *p* < 0.05 was deemed as statistically significant.

### Prediction of upstream miRNA/lncRNA of GPRIN1

The Starbase database was employed to predict miRNAs interacted with messenger RNA (mRNA) and lncRNAs acted with miRNA.

### GEPIA database analysis

GEPIA (http://gepia.cancer-pku.cn/) was utilized for survival analysis and helped evaluate the correlation between GPRIN1 and immune checkpoint expression. |R| > 0.1 and *p* < 0.05 were set to identify selection criteria of statistical significance.

### TIMER database analysis

TIMER (https://cistrome.shinyapps.io/timer/) was used to evaluate the correlation between genes and immune cells where *p* < 0.05 was considered to embody statistical significance.

### Statistical analysis

The statistical analysis in this work was performed by R package or online database. |Log2FC| > 1and *p* < 0.05 were considered to embody statistical significance.

## RESULTS

### Pan‐cancer analysis of GPRIN1

**FIGURE 1:**
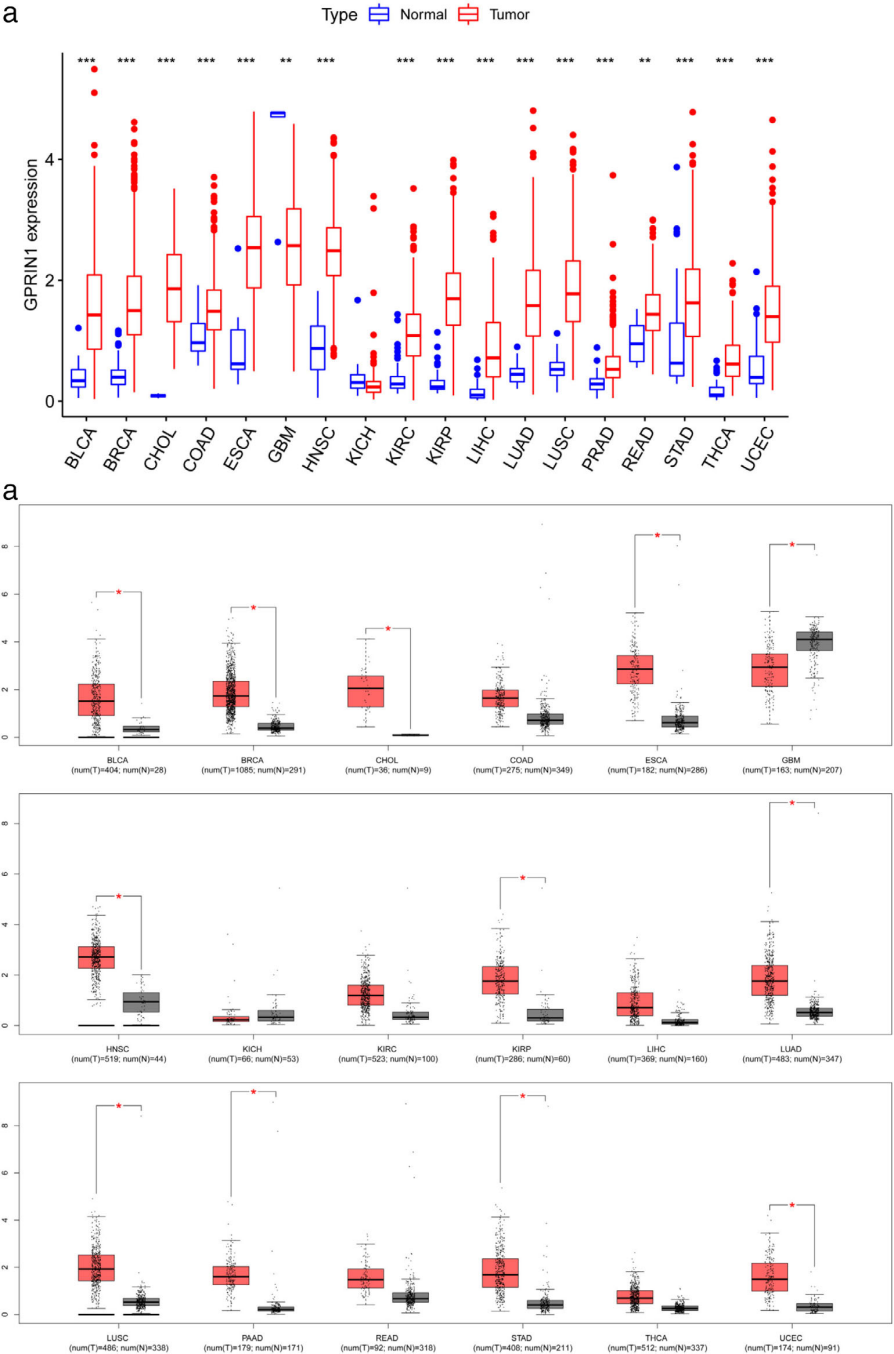
Expression analysis for GPRIN1 in multiple cancers. (a) The expression of GPRIN1 in 33 types of human cancer based on TCGA. (b) The expression of GPRIN1 in 33 types of human cancer based on TCGA and GTEx. *p value < 0.05; **p value < 0.01; ***p value < 0.001

In previous screening of tumorigenesis‐related differential genes conducted by our research group, it was found that GPRIN1 was highly expressed in tumor tissues. The pan‐cancer analysis suggested that the expression of GPRIN1 increased in 16 tumor types, that is, bladder urothelial carcinoma (BLCA), breast invasive carcinoma (BRCA), cholangiocarcinoma (CHOL), colon adenocarcinoma (COAD), esophageal carcinoma (ESCA), head and neck squamous cell carcinoma (HNSC), kidney renal clear cell carcinoma (KIRC), KIRP, liver hepatocellular carcinoma (LIHC), LUAD, lung squamous cell carcinoma (LUSC), prostate adenocarcinoma (PRAD), rectum adenocarcinoma (READ), stomach adenocarcinoma (STAD), thyroid carcinoma (THCA), and uterine corpus endometrial carcinoma (UCEC), and decreased in two tumor types, that is, glioblastoma multiforme (GMB) and kidney chromophobe (KICH) (Figure 1(a)). As shown in Figure 1(b), the expression of GPRIN1 in BLCA, BRCA, CHOL, ESCA, HNSC, KIRP, LUAD, LUSC, pancreatic adenocarcinoma (PAAD), STAD, and UCEC increased considerably, but it diminished significantly in GBM. Therefore, GPUIN1 showed an upregulation in BLCA, BRCA, CHOL, ESCA, HNSC, KIRP, LUAD, LUSC, PAAD, STAD, and UCEC, and a downregulation in GBM, indicating that GPRIN1 might play a key role in these 12 kinds of tumorigenesis.

### Effect of GPRIN1 on the prognosis of tumor

**FIGURE 2:**
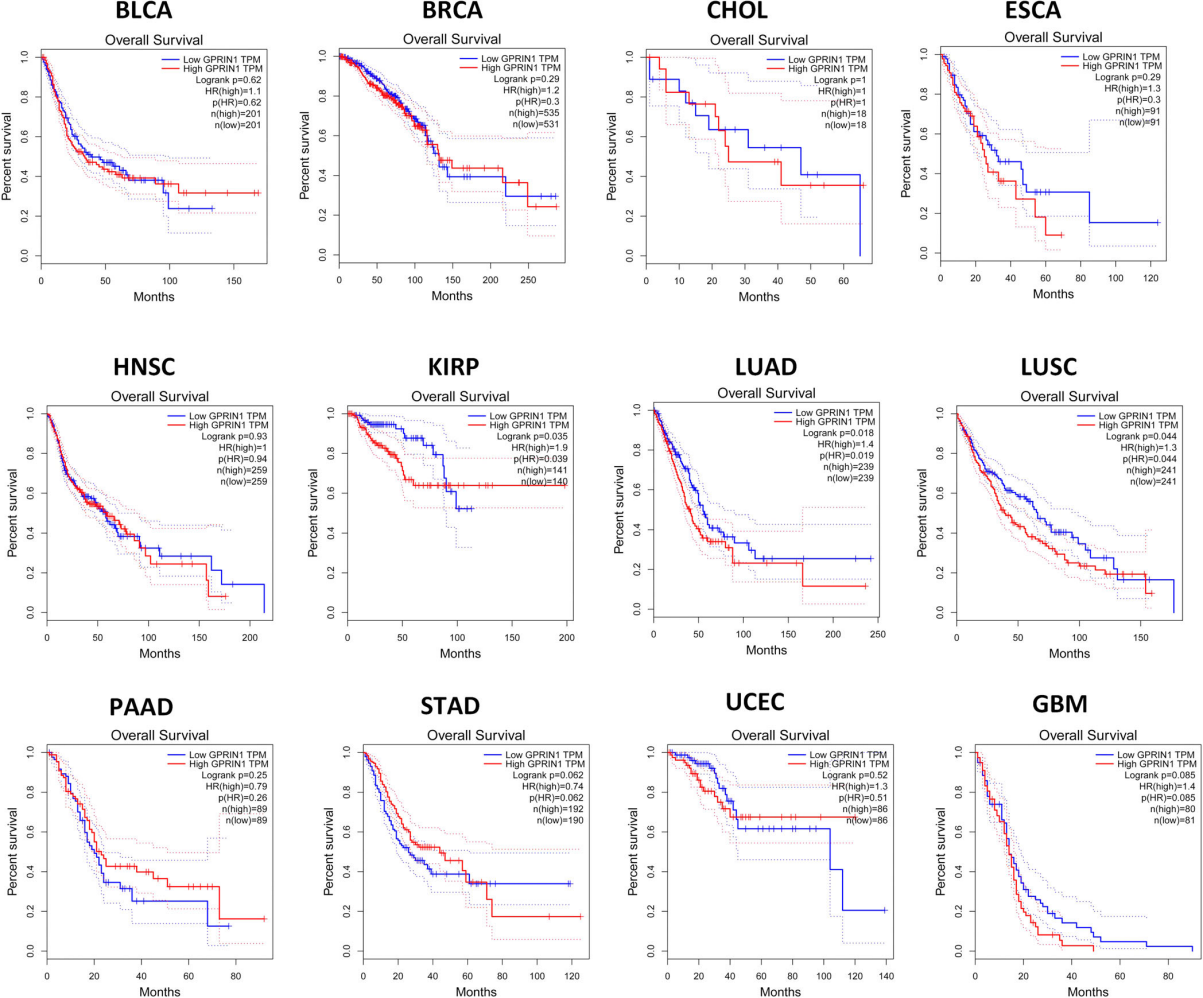
The overall survival analysis for GPRIN1 in various human cancers

Survival analysis on GPUIN1 was conducted in BLCA, BRCA, CHOL, ESCA, HNSC, KIRP, LUAD, LUSC, PAAD, STAD, UCEC, and GBM. As shown in Figure 2, the high expression in KIRP, LUAD, and LUSC was accompanied by poor prognosis, especially in KIRP and LUAD. Therefore, GPRIN1 might be a biomarker of poor prognosis in patients with KIRP and LUAD.

### Predictive analysis of upstream miRNA of GPRIN1

**FIGURE 3:**
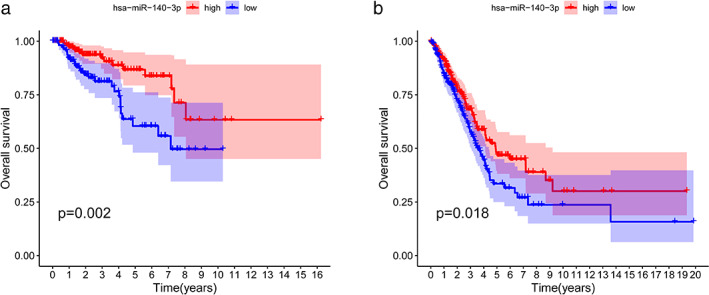
Survival analysis of miR‐140‐3p in KIRP (a) and LUAD (b)

The Starbase database was used for predicting upstream miRNAs that could regulate the expression of GPRIN1 (see Supporting Information Table S1). As there should be a negative correlation between miRNA and GPRIN1, the results of expression correlation analysis showed that six miRNAs were significantly negatively correlated with GPRIN1 in KIRP, and 13 miRNAs were negatively correlated with GPRIN1 in LUAD (Table 1). The survival analysis demonstrated that miR‐140‐3p, miR‐140‐5p, miR‐181‐5p, miR‐185‐5p, miR‐362‐3p, and miR‐1270 all showed an inverse correlation with prognosis in KIRP. miR‐181c‐5p, miR‐181d‐5p, miR‐23b‐3p, miR‐184, miR‐181a‐5p, miR‐1287‐5p, miR‐140‐3p, miR‐362‐3p, miR‐335‐5p, and miR‐628‐5p were negatively correlated with the prognosis in LUAD. miR‐140‐3p was found to be the upstream miRNA of GPRIN1 shared by KIRP and LUAD (Figure 3).

**TABLE 1:** Correlation analysis between GPRIN1 and predicted miRNAs

Cancer	Gene	miRNA	Cor	*p* value	logFC	diffPval
KIRP	GPRIN1	hsa‐miR‐140‐3p	−0.37827	4.05E‐11	−0.01796	0.97316
	GPRIN1	hsa‐miR‐185‐5p	−0.3132	6.32E‐08	0.978937	1.93E‐10
	GPRIN1	hsa‐miR‐362‐3p	−0.30888	9.72E‐08	−1.04562	1.10E‐08
	GPRIN1	hsa‐miR‐140‐5p	−0.2694	3.69E‐06	−0.65712	1.28E‐07
	GPRIN1	hsa‐miR‐1270	−0.22944	8.28E‐05	0.519935	0.007381
	GPRIN1	hsa‐miR‐181a‐5p	−0.21203	0.000292	0.277159	0.022883
LUAD	GPRIN1	hsa‐miR‐181c‐5p	−0.31979	1.64E‐13	0.267642	0.028594
	GPRIN1	hsa‐miR‐181d‐5p	−0.22866	1.83E‐07	0.646507	1.90E‐06
	GPRIN1	hsa‐miR‐23b‐3p	−0.18826	1.87E‐05	0.091887	0.173238
	GPRIN1	hsa‐miR‐184	−0.17819	5.02E‐05	−2.96043	7.92E‐21
	GPRIN1	hsa‐miR‐181a‐5p	−0.16026	0.000276	−0.0986	0.281727
	GPRIN1	hsa‐miR‐1287‐5p	−0.15835	0.000327	0.843028	3.76E‐11
	GPRIN1	hsa‐miR‐192‐5p	−0.15139	0.000596	1.708925	1.61E‐10
	GPRIN1	hsa‐miR‐1913	−0.14028	0.001462	0.049539	0.010832
	GPRIN1	hsa‐miR‐140‐3p	−0.12673	0.004099	−1.11738	8.16E‐21
	GPRIN1	hsa‐miR‐215‐5p	−0.12431	0.004873	0.854984	0.116452
	GPRIN1	hsa‐miR‐362‐3p	−0.1227	0.005434	0.182563	0.398877
	GPRIN1	hsa‐miR‐335‐5p	−0.12214	0.005677	−0.21038	0.000161
	GPRIN1	hsa‐miR‐628‐5p	−0.11783	0.00764	1.321685	2.33E‐12

### Predictive analysis of upstream lncRNAs of miR‐140‐3p

**FIGURE 4:**
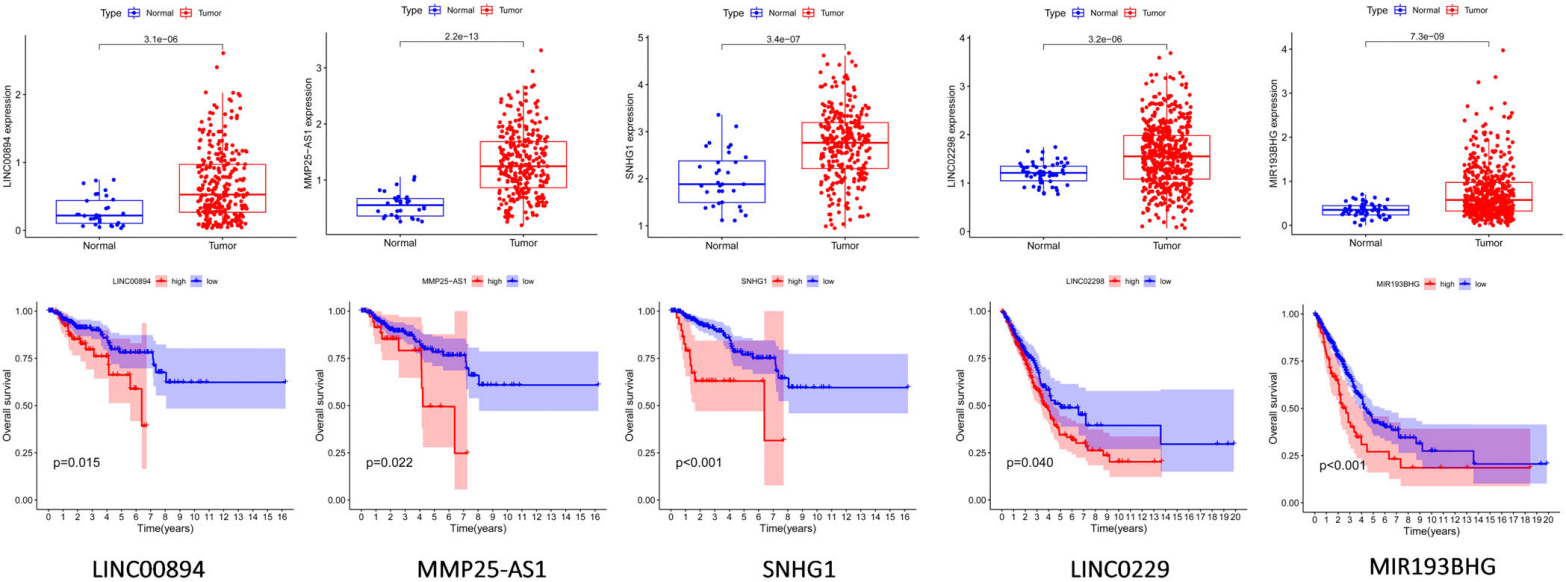
Expression analysis and survival analysis for upstream lncRNAs of miR‐140‐3p/GPRIN1 in KIRP/LUAD

The Starbase database was also employed to predict the upstream lncRNA of miR‐140‐3p and identified a total of 135 lncRNAs. Referring to the regulation mechanism of ceRNA, lncRNA should be negatively correlated with miRNA, but positively correlated with GPRIN1. The expression correlation analysis showed that LINC00894, MMP25‐AS1, N4BP2L2‐IT2, SNHG1, STAG3L5P‐PVRIG2P‐PILRB, TMEM147‐AS1, and TUG1 showed a positive correlation with GPRIN1, and a negative correlation with miR‐140‐3p in KIRP (Table 2). In LUAD, MIR193BHG was positively correlated with GPRIN1 and negatively correlated with miR‐140‐3p. The survival analysis suggested that the high expression of LINC00894, MMP25‐AS1, and SNHG1 was related to the poor prognosis of KIRP, and the high expression of MIR193BHG was related to the poor prognosis of LUAD (Figure 4). Through the construction of a ceRNA regulatory mechanism network, LINC00894, MMP25‐AS1, and SNHG1might be the upstream potential lncRNAs of the miR140‐3p/GPRIN1 axis in KIRP, and LINC02298 and MIR193BHG may be the upstream potential lncRNAs of the miR140‐3p/GPRIN1 axis in LUAD.

**TABLE 2:** Correlation analysis between lncRNA and miR‐140‐3p or GPRIN1

Cancer	lncRNA	Gene	Cor	*p* value	logFC	diffPval
KIRP	LINC02298	GPRIN1	0.111845	0.057587	6.18E‐01	3.91E‐06
		hsa‐miR‐140‐3p	−0.25518	1.20E‐05	6.18E‐01	3.91E‐06
	LINC00894	GPRIN1	0.142357	0.015501	3.89E‐01	3.07E‐06
		hsa‐miR‐140‐3p	−0.27307	2.69E‐06	3.89E‐01	3.07E‐06
	N4BP2L2‐IT2	GPRIN1	0.151988	0.00972	2.45E‐01	3.21E‐05
		hsa‐miR‐140‐3p	−0.24117	3.61E‐05	2.45E‐01	3.21E‐05
	TUG1	GPRIN1	0.276773	1.95E‐06	1.39E‐01	0.038204
		hsa‐miR‐140‐3p	−0.35788	4.91E‐10	1.39E‐01	0.038204
	STAG3L5P‐PVRIG2P‐PILRB	GPRIN1	0.182572	0.001858	6.58E‐01	9.15E‐07
		hsa‐miR‐140‐3p	−0.25963	8.36E‐06	6.58E‐01	9.15E‐07
	MMP25‐AS1	GPRIN1	0.32708	1.52E‐08	0.74897	2.20E‐13
		hsa‐miR‐140‐3p	−0.26306	6.29E‐06	0.74897	2.20E‐13
	TMEM147‐AS1	GPRIN1	0.202996	0.000529	2.92E‐01	0.004841
		hsa‐miR‐140‐3p	−0.26439	5.63E‐06	2.92E‐01	0.004841
	SNHG1	GPRIN1	0.202845	0.000534	7.07E‐01	3.36E‐07
		hsa‐miR‐140‐3p	−0.21365	0.000261	7.07E‐01	3.36E‐07
	OIP5‐AS1	GPRIN1	0.081594	0.166452	1.30E‐01	0.032281
		hsa‐miR‐140‐3p	−0.2322	7.06E‐05	1.30E‐01	0.032281
LUAD	LINC02298	GPRIN1	−0.07345	0.096893	0.361085	3.22E‐06
		hsa‐miR‐140‐3p	−0.13682	0.001931	0.361085	3.22E‐06
	MIR193BHG	GPRIN1	0.162016	0.000232	0.384463	7.34E‐09
		hsa‐miR‐140‐3p	−0.13226	0.002712	0.384463	7.34E‐09
	SNHG1	GPRIN1	0.036109	0.414764	1.309259	2.11E‐27
		hsa‐miR‐140‐3p	−0.17179	9.57E‐05	1.309259	2.11E‐27
	UBA6‐AS1	GPRIN1	0.083112	0.060226	0.40373	1.64E‐22
		hsa‐miR‐140‐3p	−0.16552	0.000172	0.40373	1.64E‐22

### GPRIN1 and immune cell infiltration

**FIGURE 5:**
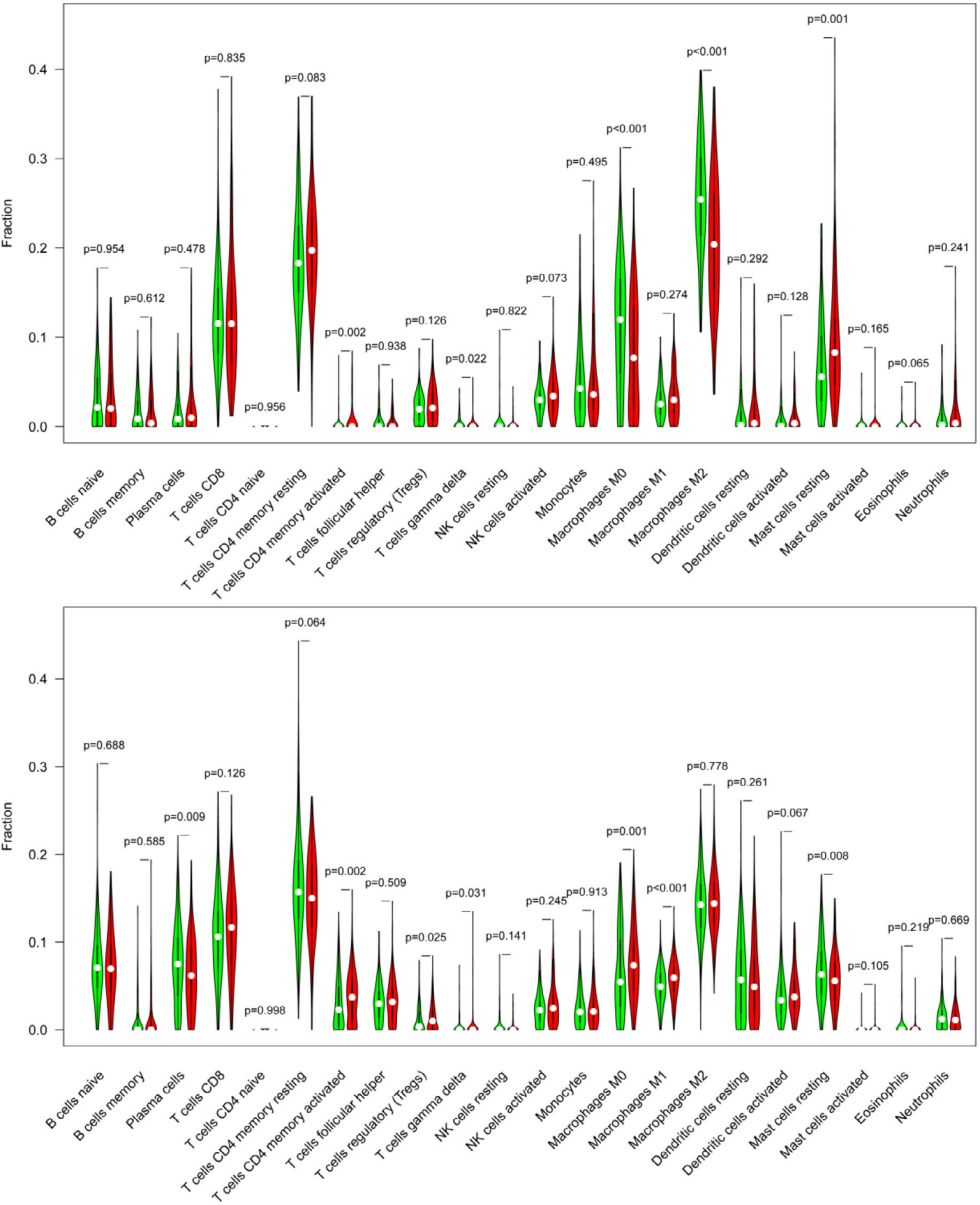
The relationship of immune cell infiltration with GPRIN1 level in KIRP (a) and LUAC (b)

As shown in Figure 5, there were significant differences in activated memory CD4 T cells, γδ T cells, M0 macrophages, M2 macrophages, and mast cells resting between the high‐expressed and low‐expressed GPRIN1 groups in KIRP. There were considerable differences in plasma cells, activated memory CD4 T cells, regulatory T cells (Tregs), γδ T cells, M0 macrophages, M1 macrophages, and resting mast cells between the high‐expressed and low‐expressed GPRIN1 groups in LUAD. In KIRP, the expression of GPRIN1 proved to be negatively correlated with M0 macrophages, M2 macrophages, activated mast cells and monocytes, but positively correlation with mast cells resting, activated NK cells, and activated memory CD4 T cells. In LUAD, GPRIN1 was negatively correlated with resting mast cells, plasma cells, and γδ T cells, and was positively correlated with activated dendritic cells, M0 macrophages, M1 macrophages, activated mast cells, and activated memory CD4 T cells.

### Correlation between GPRIN1 and expression of immune cell biomarkers

As shown in Table 3, GPRIN1 had a positive correlation with the biomarker expressions of B cells, CD8+ T cells, and M1 macrophages, and was negatively correlated with M2 macrophages in KIRP. In LUAD, GPRIN1 showed another positive correlation with CD8+ T cells, CD4+ T cells, M1 macrophages, and M2 macrophages, and negative correlation with B cells.

**TABLE 3:** Correlation analysis between GPRIN1 and immune cell biomarker

Cancer	Gene 1	Immune cell	Gene	Cor	*p* value
KIRP	GPRIN1	B cell	CD19	0.017350544	0.768984828
	GPRIN1	B cell	CD79A	0.049740982	0.399311108
	GPRIN1	CD8+ T cell	CD8A	0.073773018	0.211015282
	GPRIN1	CD8+ T cell	CD8B	0.018041304	0.759924831
	GPRIN1	CD4+ T cell	CD4	−0.12054548	0.040624999
	GPRIN1	M1 macrophage	NOS2	0.080583662	0.171873699
	GPRIN1	M1 macrophage	IRF5	0.121136101	0.039645399
	GPRIN1	M1 macrophage	PTGS2	0.011511653	0.845413995
	GPRIN1	M2 macrophage	CD163	−0.149456111	0.011016356
	GPRIN1	M2 macrophage	VSIG4	−0.098806328	0.093623328
	GPRIN1	M2 macrophage	MS4A4A	−0.186455475	0.001476748
	GPRIN1	Neutrophil	CEACAM8	0.082684786	0.160929833
	GPRIN1	Neutrophil	ITGAM	0.039498071	0.503386444
	GPRIN1	Neutrophil	CCR7	−0.008869268	0.880593827
	GPRIN1	Dendritic cell	HLA‐DPB1	−0.190670366	0.001145572
	GPRIN1	Dendritic cell	HLA‐DQB1	−0.144514378	0.013993436
	GPRIN1	Dendritic cell	HLA‐DRA	−0.198481685	0.000705705
	GPRIN1	Dendritic cell	HLA‐DPA1	−0.177619516	0.002472826
	GPRIN1	Dendritic cell	CD1C	0.048246536	0.413864168
	GPRIN1	Dendritic cell	NRP1	0.307573679	1.11E‐07
	GPRIN1	Dendritic cell	ITGAX	−0.050524997	0.391914787
LUAD	GPRIN1	B cell	CD19	−0.048904656	0.262783191
	GPRIN1	B cell	CD79A	−0.048021876	0.271504287
	GPRIN1	CD8+ T cell	CD8A	0.095932559	0.027838374
	GPRIN1	CD8+ T cell	CD8B	0.030342515	0.487306175
	GPRIN1	CD4+ T cell	CD4	0.061879207	0.156389107
	GPRIN1	M1 macrophage	NOS2	0.133319714	0.002199987
	GPRIN1	M1 macrophage	IRF5	0.279212209	8.58E‐11
	GPRIN1	M1 macrophage	PTGS2	0.070376858	0.106896269
	GPRIN1	M2 macrophage	CD163	0.116259273	0.007634581
	GPRIN1	M2 macrophage	VSIG4	0.07081396	0.104731688
	GPRIN1	M2 macrophage	MS4A4A	0.038235675	0.381367227
	GPRIN1	Neutrophil	CEACAM8	−0.120996573	0.005458827
	GPRIN1	Neutrophil	ITGAM	0.133700664	0.002136111
	GPRIN1	Neutrophil	CCR7	−0.029815617	0.494901421
	GPRIN1	Dendritic cell	HLA‐DPB1	−0.056058676	0.199199354
	GPRIN1	Dendritic cell	HLA‐DQB1	0.018598546	0.670318849
	GPRIN1	Dendritic cell	HLA‐DRA	−0.026250274	0.547910733
	GPRIN1	Dendritic cell	HLA‐DPA1	−0.00237809	0.956593502
	GPRIN1	Dendritic cell	CD1C	−0.109818668	0.011758213
	GPRIN1	Dendritic cell	NRP1	0.093725112	0.031652503
	GPRIN1	Dendritic cell	ITGAX	0.108966561	0.012430503

### The relationship between GPRIN1 and immune checkpoints

**FIGURE 6:**
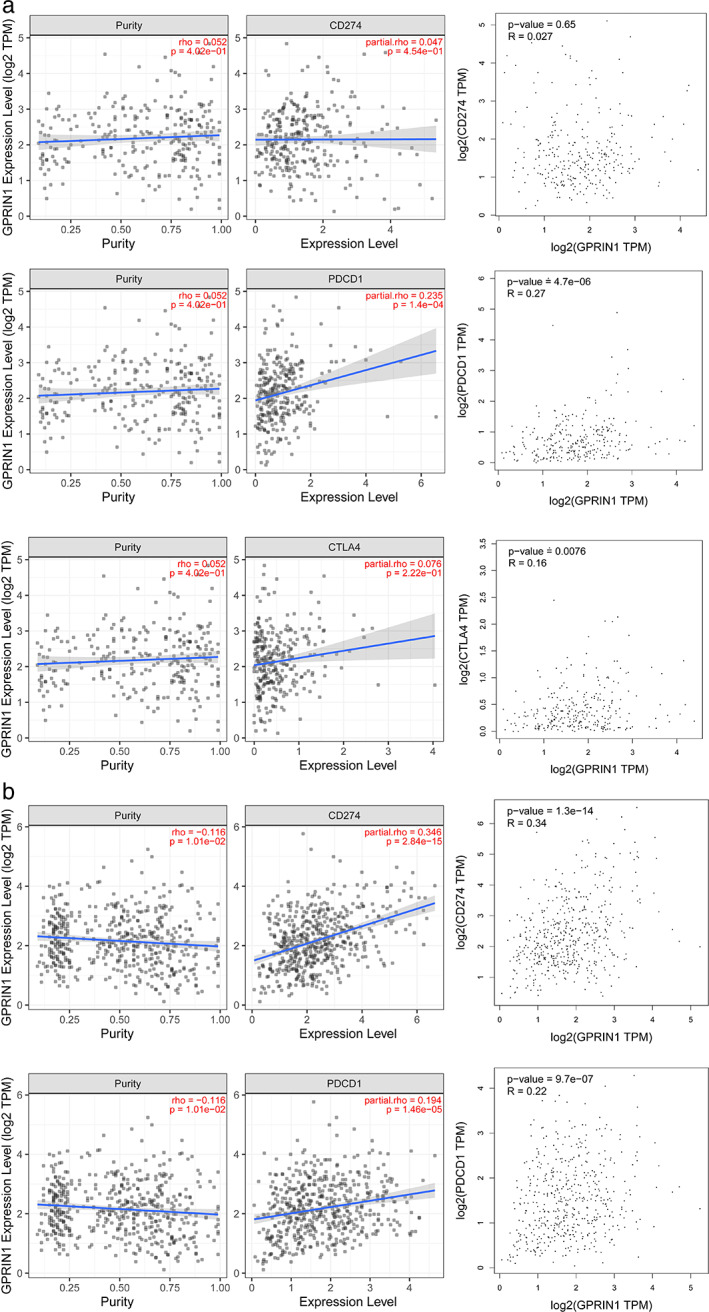
Correlation of GPRIN1 expression with PD‐1, PD‐L1, and CTLA‐4 expression in KIRP and LUAD

PD1, PD‐L1, and CTLA‐4 are important immune checkpoints responsible for tumor immune escape. The relationship between GPRIN1 and PD1, PD‐L1, and CTLA‐4 was evaluated. As shown in Figure 6, significant positive correlations between GPRIN1 and PD1, PD‐L1 and CTLA‐4 in LUAD, and GPRIN1 and PD1 in KIRP were found.

## DISCUSSION

With still no effective treatment for tumors, cancer remain one of the leading causes of human death worldwide. By elucidating the molecular mechanism relatable to tumors, it is possible that important clues for developing effective therapeutic targets or specific prognostic biomarkers may be sought out. Currently, studies have found that tumorigenesis concerns major molecules and signal pathways. In recent years, the wide application of bioinformatics has helped to find molecular pathways that are closely related to tumors. In this work, differentiated expression analysis was made on 33 types of tumors based on TCGA database, and GPRIN1 was proved to be significantly overexpressed in KIRP and LUAD with relation to poor prognosis.

It has been reported that lncRNA can regulate gene expression through the ceRNA mechanism. Ninety‐one upstream miRNAs were predicted to bind to GPRIN1 by using the Starbase database, and expression correlation and survival analysis have suggested that six miRNAs were relatable to better prognosis in KIRP. Ten miRNAs were associated with better prognosis in LUAD. It was reported that such miRNAs could act as tumor suppressors during tumorigenesis. mir‐140‐3p plays an inhibitory role in lung adenocarcinoma, non‐small‐cell lung cancer and lung squamous cell carcinoma.^2^, ^3^, ^4^ miR‐140‐5p was found to inhibit the proliferation and metastasis of renal clear cell carcinoma by targeting Insulin Like Growth Factor 1 Receptor (IGF1R).^5^ miR‐185‐5p could suppress tumor malignancy of lung adenocarcinoma,^6^ and function as a tumor suppressor in metastatic clear‐cell renal carcinoma by targeting HIF‐2α.^7^ Downregulation of lncRNA LUCAT1 could suppress the migration and invasion of bladder cancer by targeting miR‐181c‐5p.^8^ microRNA‐181d‐5p was proved to have tumor‐suppressive effects on non‐small‐cell lung cancer through the CDKN3‐mediated Akt signaling pathway.^9^ miR‐23b‐3p was significantly associated with tumor size, depth of invasion, liver metastasis, and Tumor‐Node‐Metastasis (TNM) stage of non‐small‐cell lung cancer.^10^ miR‐140‐3p was found to be related to the prognosis of KIRP and LUAD in this study.

Based on the ceRNA hypothesis, the upstream regulatory lncRNA of the miR‐140‐3p/GRPIN1 axis is supposed to be the carcinogenic lncRNA of KIRP/LUAD, so the Starbase database was used to predict the upstream lncRNA of the miR‐140‐3p/GRPIN1 axis. Through expression analysis, survival analysis, and correlation analysis, it was proposed that LINC00894, MMP25‐AS1, and SNHG1 might be the regulatory lncRNAs of the miR140‐3p/GPRIN1 axis in KIRP, and LINC02298 and MIR193BHG the regulatory lncRNAs of the upstream potential of the miR140‐3p/GPRIN1 axis in LUAD. It has been reported that LINC00894 could enhance the progression of breast cancer by regulating ZEB1 expression through sponging miR‐429.^11^ SNHG1 was reported to be able to promote renal cell carcinoma progression and metastasis by reversely regulating miR‐137.^12^ Therefore, the LncRNA‐miR140‐3p‐GPRIN1 axis might serve as the potential regulatory pathway of KIRP/LUAD.

A large number of studies have confirmed that tumor immune cell infiltration can affect the efficacy of chemotherapy and immunotherapy, and the prognosis of patients.^13^, ^14^, ^15^ Our work has shown that there does exist a significant positive correlation between GPRIN1 and CD4 + T cell, M1macrophage infiltration, and GPRIN1 is also positively correlated with the biomarkers of these infiltrating immune cells. Therefore, immune cell infiltration may be involved in the occurrence and development of GPRIN1‐mediated KIRP/LUAD. As immunotherapy shows a close correlation with immune checkpoints, the relation between GPRIN1 and immune checkpoints was evaluated. On top of that, GPRIN1 displayed a close correlation with PD1, PD‐L1 or CTLA‐4 in KIRP and LUAD, suggesting that targeting GPRIN1 may improve the effect of immunotherapy.

To conclude, this study has found that GPRIN1, with poor prognosis of KIRP and LUAD, is highly expressed in various types of human tumors. The upstream regulatory mechanism of GPRIN1 was defined and the regulatory network of LINC00894/MMP25‐AS1/SNHG1/LINC02298/MIR193BHG‐miR‐140‐3p‐GPRIN1 was built. GPRIN1 also could affect the outcome of cancer treatment by affecting tumor immune cell infiltration and immune checkpoint expression. However, limitations still pervade as retrospective analysis collected data from public databases, which makes bias and inadequacy inevitable. Therefore, larger scales of prospective studies are needed to further verify the validity of the prognostic features.

## CONFLICT OF INTEREST

The authors report no competing interests in this work.

## Supporting information

**Supporting Information Table S1** Predicting the upstream miRNAs of GPRIN1 using the Starbase database
**Supporting Information Table S2** Predicting lncRNAs of miR‐140‐3p using the Starbase database

## References

[1] Lee H, Palm J, Grimes SM, Ji HP. The cancer genome atlas clinical explorer: a web and mobile interface for identifying clinical‐genomic driver associations. Genome Med. 2015;7:112. 26507825 10.1186/s13073-015-0226-3 PMC4624593

[2] Wang Y, Wo Y, Lu T, Sun X, Liu A, Dong Y, et al. Circ‐AASDH functions as the progression of early stage lung adenocarcinoma by targeting miR‐140‐3p to activate E2F7 expression. Transl Lung Cancer Res. 2021;10:57–70. 33569293 10.21037/tlcr-20-1062 PMC7867743

[3] Wu S, Wang H, Pan Y, Yang X, Wu D. miR‐140‐3p enhances cisplatin sensitivity and attenuates stem cell‐like properties through repressing Wnt/beta‐catenin signaling in lung adenocarcinoma cells. Exp Ther Med. 2020;20(2):1664–74. 32765679 10.3892/etm.2020.8847 PMC7388557

[4] Hu C, Zou Y, Jing LL. miR‐140‐3p inhibits progression of non‐small cell lung cancer by targeting Janus kinase 1. J Biosci. 2020;45:48. 32345774

[5] Zhang YJ, Lu C. Long non‐coding RNA HCP5 promotes proliferation and metastasis of clear cell renal cell carcinoma via targeting miR‐140‐5p/IGF1R pathway. Eur Rev Med Pharmacol Sci. 2020;24:2965–75. 32271414 10.26355/eurrev_202003_20661

[6] Li Y, Hu Y, Wu Y, Zhang D, Huang D. LINC00205 promotes tumor malignancy of lung adenocarcinoma through sponging miR‐185‐5p. Lab Med. 2021. 10.1093/labmed/lmab041 34270733

[7] Kinget L, Roussel E, Verbiest A, Albersen M, Rodríguez‐Antona C, Graña‐Castro O, et al. MicroRNAs targeting HIF‐2alpha, VEGFR1 and/or VEGFR2 as potential predictive biomarkers for VEGFR tyrosine kinase and HIF‐2alpha inhibitors in metastatic clear‐cell renal cell carcinoma. Cancers (Basel). 2021;13(12):3099. 34205829 10.3390/cancers13123099 PMC8235409

[8] Chen Y, Zhang W, Shen L, Kadier A, Huang J, Wang R, et al. Downregulation of long noncoding RNA LUCAT1 suppresses the migration and invasion of bladder cancer by targeting miR‐181c‐5p. Biomed Res Int. 2020;2020:4817608. 33282949 10.1155/2020/4817608 PMC7685804

[9] Gao LM, Zheng Y, Wang P, Zheng L, Zhang WL, Di Y, et al. Tumor‐suppressive effects of microRNA‐181d‐5p on non‐small‐cell lung cancer through the CDKN3‐mediated Akt signaling pathway in vivo and in vitro. Am J Physiol Lung Cell Mol Physiol. 2019;316:L918–33. 30628487 10.1152/ajplung.00334.2018

[10] Wang J, Xue H, Zhu Z, Gao J, Zhao M, Ma Z. Expression of serum exosomal miR‐23b‐3p in non‐small cell lung cancer and its diagnostic efficacy. Oncol Lett. 2020;20:30. 32774503 10.3892/ol.2020.11891 PMC7405601

[11] Meng DF, Shao H, Feng CB. LINC00894 enhances the progression of breast cancer by sponging miR‐429 to regulate ZEB1 expression. Onco Targets Ther. 2021;14:3395–407. 34079285 10.2147/OTT.S277284 PMC8164724

[12] Zhao S, Wang Y, Luo M, Cui W, Zhou X, Miao L. Long noncoding RNA small nucleolar RNA host gene 1 (SNHG1) promotes renal cell carcinoma progression and metastasis by negatively regulating miR‐137. Med Sci Monit. 2018;24:3824–31. 29874202 10.12659/MSM.910866 PMC6018379

[13] Jiang Q, Sun J, Chen H, Ding C, Tang Z, Ruan Y, et al. Establishment of an immune cell infiltration score to help predict the prognosis and chemotherapy responsiveness of gastric cancer patients. Front Oncol. 2021;11:650673. 34307129 10.3389/fonc.2021.650673 PMC8299334

[14] Zhou P, Lu Y, Zhang Y, Wang L. Construction of an immune‐related six‐lncRNA signature to predict the outcomes, immune cell infiltration, and immunotherapy response in patients with hepatocellular carcinoma. Front Oncol. 2021;11:661758. 34277410 10.3389/fonc.2021.661758 PMC8283691

[15] Yuan C, Xiang L, Cao K, Zhang J, Luo Y, Sun W, et al. The prognostic value of tumor mutational burden and immune cell infiltration in esophageal cancer patients with or without radiotherapy. Aging (Albany NY). 2020;12:4603–16. 32165590 10.18632/aging.102917 PMC7093160

